# A dimensionless ordered pull-through model of the mammalian lens epithelium evidences scaling across species and explains the age-dependent changes in cell density in the human lens

**DOI:** 10.1098/rsif.2015.0391

**Published:** 2015-07-06

**Authors:** Jun Jie Wu, Weiju Wu, Frederique M. Tholozan, Christopher D. Saunter, John M. Girkin, Roy A. Quinlan

**Affiliations:** 1 Biophysical Sciences Institute and School of Engineering and Computing Sciences, Durham University, Durham DH1 3LE, UK; 2 Biophysical Sciences Institute and School of Biological and Biomedical Sciences, Durham University, Durham DH1 3LE, UK; 3 Biophysical Sciences Institute and Department of Physics, Durham University, Durham DH1 3LE, UK

**Keywords:** eye lens, cell proliferation, ageing, cataract, mathematical model, scaling

## Abstract

We present a mathematical (ordered pull-through; OPT) model of the cell-density profile for the mammalian lens epithelium together with new experimental data. The model is based upon dimensionless parameters, an important criterion for inter-species comparisons where lens sizes can vary greatly (e.g. bovine (approx. 18 mm); mouse (approx. 2 mm)) and confirms that mammalian lenses scale with size. The validated model includes two parameters: *β*/*α*, which is the ratio of the proliferation rate in the peripheral and in the central region of the lens; and *γ*_GZ_, a dimensionless pull-through parameter that accounts for the cell transition and exit from the epithelium into the lens body. Best-fit values were determined for mouse, rat, rabbit, bovine and human lens epithelia. The OPT model accounts for the peak in cell density at the periphery of the lens epithelium, a region where cell proliferation is concentrated and reaches a maximum coincident with the germinative zone. The *β*/*α* ratio correlates with the measured FGF-2 gradient, a morphogen critical to lens cell survival, proliferation and differentiation. As proliferation declines with age, the OPT model predicted age-dependent changes in cell-density profiles, which we observed in mouse and human lenses.

## 1. Introduction

Vision is one of the most important senses for survival and the eye and the eye lens have evolved in ways that are linked closely with an animal's environment. In particular, the lens integrates cell structure with tissue form to produce a graded refractive index that reduces spherical aberration to enhance vision in animals [1]. The lens grows throughout life [2] and is a tissue in which both biological and physical forces combine in its formation. It is only now that the interplay between these is being explored. Here we bring together previously published results and new measurements through a mathematical model of the mammalian lens epithelium applicable to different mammalian species independent of their age.

**Figure 1.**
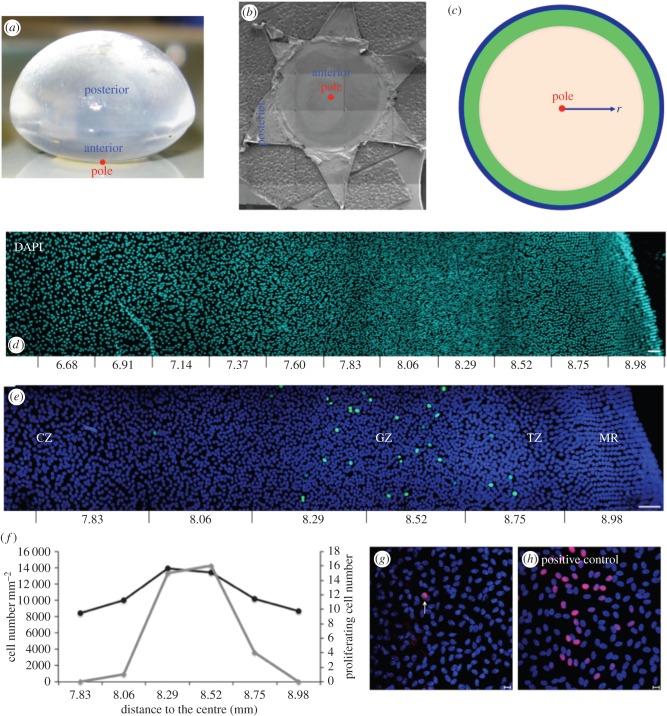
Relationship of lens morphology to its epithelium and the schematic of the mathematical model. Biological images showing cell-density variation. (*a*) Bovine lens showing its anterior and posterior surfaces and the location of the anterior pole (red circle). (*b*) Dissected and flat-mounted lens capsule. The anterior pole (red circle) is indicated. (*c*) Schematic of the mathematical model indicating the pole (red circle) and, for orientation purposes, the different zones; CZ (beige); GZ (green) and TZ/MR (blue). The origin of radial distance *r* from the anterior pole is indicated. (*d*) A montage of images taken from a flat-mounted bovine lens epithelium stained with DAPI to identify cell nuclei. The montage illustrates the cell-density changes in the lens periphery (equator). Distance (mm) from the anterior pole is indicated. Scale bar, 50 μm. (*e*) Flat-mounted bovine lens epithelium stained with the cell proliferation marker Ki-67 (green channel) and DAPI (blue channel). The zone with the most Ki-67 labelling is defined in the literature as the GZ, which is in the lens periphery. The MR is the most peripheral feature of the lens epithelium. The TZ is between the MR and GZ. Distance (mm) from the anterior pole is indicated. Scale bar, 50 μm. (*f*) Cell density (black line) and Ki-67 labelling (grey line) from (*c*) were counted confirming that most Ki-67 labelling coincided with the highest cell density in the lens epithelium. (*g*) TUNEL staining of the bovine lens epithelium (red channel) and counter-stained with DAPI (blue channel). TUNEL-positive cells are rarely observed. One example of a TUNEL-positive cell is shown (white arrow). Scale bar, 10 μm. (*h*) A flat-mounted epithelium was exposed to Benzonase Nuclease (100 U ml^−1^) as a positive control to introduce more DNA strand breaks prior to TUNEL staining. Scale bar, 10 μm.

The eye lens is contained within a thick basement membrane called the lens capsule (figure 1). A monolayer of polarized epithelial cells grows on the inner anterior surface of the lens capsule [3], with their apical ends facing the lens interior and contacting the apical ends of the underlying fibre cells that are part of the lens cortex, an interaction that regulates epithelial cell proliferation [4,5]. Although the eye is growing one can envisage at any particular age a population balance at every radial position where the net production of cells is balanced by their migration towards the periphery. The bulk of the lens comprises fibre cells that have differentiated from epithelial cells [6], a process that starts in a region of the lens epithelium [7,8]) termed the transitional zone (TZ) at the lens periphery which includes the meridional rows (MR) in animal lenses [9,10]. The TZ and MR comprise the most distal region of the lens epithelium, with the MR being a distinct, morphological feature at the perimeter of the flat-mounted epithelium [11]. Epithelial cells proliferate throughout the lens epithelium albeit rarely in the MR [11]; although the mitotic index varies considerably, it peaks in a region close to the lens equator that is identified in the literature as the germinative zone (GZ; [11–14]).

Previous studies have established that progeny resulting from cell division in the GZ move into the more equatorial TZ [8,14], a zone where cells prepare to exit the epithelium via the MR [8,9,14] and form the lens modiolus [15]. Some consider the cells in the MR to be the earliest fibre cells because of the cell elongation [16], but they serve as a very distinct feature marking the extremity of the lens epithelium. The modiolus is where the tapered apical ends of equatorial cortical fibre cells interact with the apical ends of the epithelial cells and is immediately adjacent to the MR. This has also been referred to as the lens modiolus or fulcrum [15,17], through which lens epithelial cells must pass to complete their transition into lens fibre cells. As part of this transition, the cells adopt a hexagonal profile and neighbours are appropriately interdigitated, a feature that contributes to lens geometry [9,18].

Cell proliferation rates of lens epithelial cells are age-dependent and they decline as adult animals age, for example, rabbit and mouse [19,20]. In the human, lens epithelial cells exhibit decreased responsiveness to the mitogenic growth factor, FGF-2 [21]. This correlates with a reported age-dependent decline in central zone (CZ) cell density for human [22]. In other primates, there is also an age-dependent decline in epithelial cell density in both the CZ and GZ of lenses from older compared with younger monkeys [23]. A decline in cell density with age has also been reported for rats [24]. Therefore, lens epithelial cell proliferation and cell density appear to be a function of age, but so far no attempt has been made to mathematically model these age-dependent changes.

Previous mathematical models of the eye lens have concentrated on either lens shape [25–28], or fibre cell organization [29], or lens physiology [30] or thermal modelling [31]. A recently developed a multi-parameter model [32] uses stochastic-based modelling and artificially assigned boundaries to account for epithelial cell differentiation in the mouse epithelium. Our model is based upon linear differential equations and requires only three parameters, which are all dimensionless. The model is therefore independent of lens size and the dimensionless feature means that our model provides the basis for predicting the epithelial cell-density changes that accompany lens ageing and is applicable to lenses varying greatly in size from different mammals.

## 2. Material and methods

### 2.1. Eye lens materials

Lenses were collected from up to 30-month-old cows (figure 1*a*), four-month-old rats, six-month-old rabbits and different aged mice (five and six weeks, 3, 12, 19, 24 and 35 months). At least three lens pairs were analysed per species and age group.

Human lenses donated for corneal transplantation were obtained from the Bristol Eye Bank (UK). Eyes were obtained 24–36 h post-mortem. Thirty-eight eyes from 24 donors (58 ± 21 years old; age range: 20–89 years old) were divided into four age groups that were gender neutral for cell-density measurements: 20–30 (six eyes, 24.5 ± 4.2 years old), 40–50 (11 eyes, 44.5 ± 1.6 years old), 60–70 (eight eyes, 61.8 ± 2.7 years old) and 80–90 (13 eyes, 83.7 ± 5.3 years old) years old. Some were also used to measure age-dependent changes in cell proliferation. Age-dependent apoptosis was measured and compared between two groups (six eyes, 45.3 ± 0.58 years old and eight eyes, 69.5 ± 12 years old).

### 2.2. Immunofluorescence microscopy

Here we used a flat-mounting technique (figure 1*b*; [8,9,11,22,24,33]) rather than attempting to count the cells in intact lenses [16]. This avoids the potential to distort the lens as it is held in a wedged holder prior to imaging and the risk of distorting images at points of high curvature, for example, the mouse lens. With flat-mounting, the lens capsule could be stretched as the anterior lens surface is flattened (see the electronic supplementary material). The MR, however, appeared more distinct using the flat-mounting approach and it also facilitated lens epithelium measurements.

Lenses (figure 1*a*) were dissected and epithelium flat-mounts prepared (figure 1*b*) and then fixed in 4% (w/v) paraformaldehyde (PFA) for 20 min [34]. The cells were then permeablized in 0.05% (v/v) Triton X-100 and blocked in 1% (w/v) bovine serum albumin for 30 min, followed by an incubation with primary and secondary antibodies. Primary antibodies comprised Ki-67 mouse monoclonal antibody (Dako, Denmark), ZO-1 rabbit polyclonal antibody (Invitrogen, USA), N-cadherin mouse monoclonal antibodies (BD Transduction Laboratories, USA). Secondary antibodies, drug and vital dye comprised goat anti-mouse FITC, goat anti-rabbit TRITC secondary antibodies, FITC-phalloidin (Sigma-Aldrich, USA) and DAPI (Molecular Probes Inc., USA). Flat-mounts were examined using a Zeiss LSM 510 (Carl Zeiss Ltd, Cambridge, UK).

Epithelial cell densities were measured from the MR to the anterior pole for three separate image series per flat-mount. The nuclear number per defined area was measured using ‘Delineator’, a software tool developed for this study (§2.4; electronic supplementary material). Student's *t*-test was used to analyse the cell-density measurements. A *p*-value ≤ 0.05 indicated statistically significant differences. Cell proliferation indices were measured by Ki-67 staining [35].

### 2.3. TUNEL assay

An *in situ* cell-death detection kit, TMR red (Roche Diagnostics GmbH, Germany) was used to detect cell death in the lens epithelium.

### 2.4. Delineator: lens nuclei counting algorithm

DAPI-labelled nuclei number was measured using an in-house written Python-based package ‘Delineator’. Commercial and free (e.g. ImageJ) packages were unable to reliably count cell nuclei, and manual counting was inefficient for our sample sizes. The image analysis pipeline is presented (electronic supplementary material). The method segmented even very close apposed nuclei.

## 3. Data collection to establish model parameters

### 3.1. Epithelial cell density: variation across the bovine lens epithelium

Lens epithelial nuclear (cell) densities in flat-mounted samples (figure 1*b*) were essentially constant within the central part of the CZ (figure 1*c*) but increased slowly in the distal part of CZ (a region termed pre-GZ or PGZ elsewhere [11,32]). At the lens periphery itself, a dramatic increase before a decline in cell density was observed as the MR are reached, which represent the most distal part of the flat-mounted epithelium (figure 1*d,e*). Coincident with this increased cell density in this region was increased cell length and reduced cross-section (see the electronic supplementary material). Cell proliferation (figure 1*e,f*) and apoptosis (figure 1*g,h*) were detected using Ki-67 and TUNEL staining, respectively. We measure distance from the anterior pole (figure 1*c–e*) and relate this to the CZ, GZ, TZ and MR (e.g. figure 1*e*) as commonly used descriptors found in the literature, for example [9–11,32,36], to designate particular regions of the lens epithelium. The distance between the anterior pole and the MR can be measured accurately (figure 1*c–e*). These are therefore the two reference points. Cell-density profiles were also measured for human, rabbit, rat and mouse lenses (see below).

### 3.2. Lens epithelial cell proliferation index

Most Ki-67 staining was observed in a narrow region located at the periphery of the epithelial whole-mounts of bovine lenses (figure 1*f*). This zone has been previously identified as the GZ [11,14]. Some Ki-67-positive cells were also detected in the CZ of young lenses (data not shown). The proliferation index was measured to be 0.0039 ± 0.0008 in the 30-month-old bovine, 0.01 ± 0.0018 in six-month-old rabbit, 0.0042 ± 0.0012 in 33–46-year-old human and 0.0022 ± 0.001 in 80–98-year-old human.

### 3.3. Epithelial cell apoptosis

All TUNEL-positive nuclei we observed had classic apoptotic-like morphologies including condensed chromatin and smaller fragmented nuclei. Apoptotic cells in bovine lens epithelium were rarely observed (e.g. figure 1*g*). The number of apoptotic cells in each of the 14 human lenses was very low, ranging from 0 to 23 per lens; there are *ca* 500 000 cells per lens. Apoptotic cells were not restricted to any particular region of the lens. No significant difference was present (*p* > 0.05) between middle-aged (45–46 years old) and older people (59–81 years old), suggesting that cell apoptosis does not change significantly with age for human lenses. Cell apoptosis in the bovine lens epithelia was found to be extremely rare with only one apoptotic cell being found amid several samples.

### 3.4. Comparison of cell-density changes across the lens epithelia of different mammals

In all animal lens samples examined, the cell density (number per unit area) showed a characteristic pattern, rising from a plateau in the CZ to a very distinct peak at the periphery of the epithelium, coinciding with where the most proliferating cells are found, i.e. by definition the GZ [11,32]. This peak then decayed to an intermediate cell density. The absolute cell-density values differed between species, ranging from 3500 cells mm^−2^ in rabbits to 5300 cells mm^−2^ in mice at the anterior pole and CZ, and peaking at 8650 cells mm^−2^ (human) to 13 400 cells mm^−2^ in mice and 15 900 cells mm^−2^ in rats.

### 3.5. Dimensionless analysis showing species-independent behaviour

To compare and analyse data from the lenses of different species, we developed a novel dimensionless approach and used calculated boundaries based upon the cell-density profile itself in order to produce the model.

**Figure 2.**
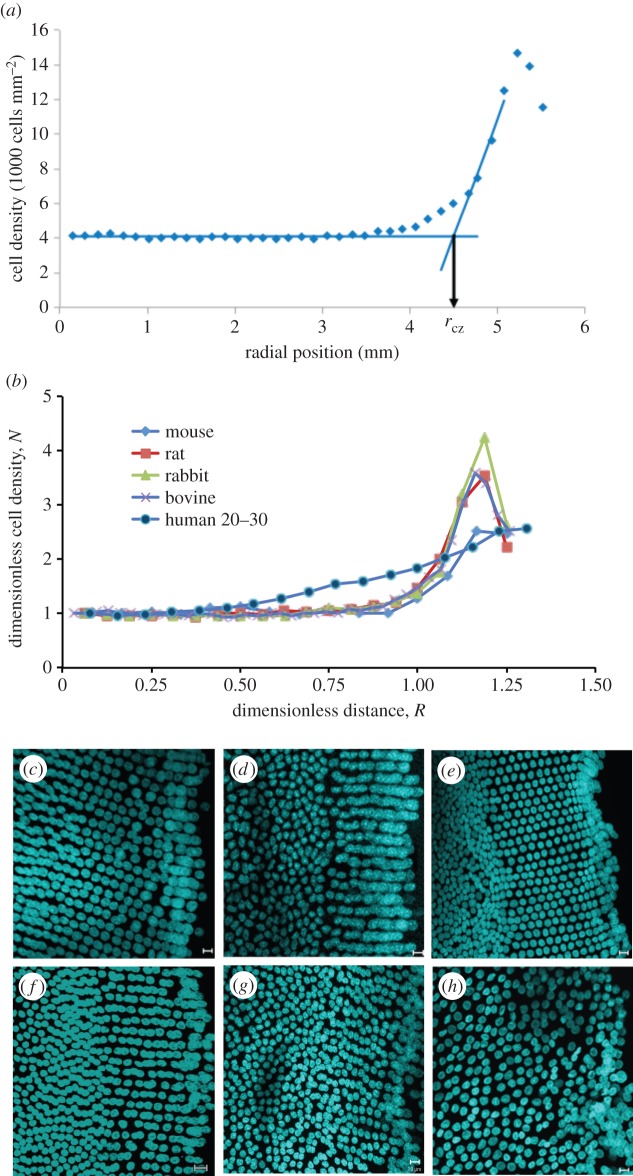
Dimensionless analysis of the spatial variation of cell density in the lens epithelium of various mammals and differences in the organization of the meridional rows (MR). (*a*) Illustration of method used to determine *r*_cz_, the distance from the pole to the beginning of GZ. (*b*) Variation of normalized epithelial cell density, *N*, with dimensionless distance, *R*, from the lens anterior pole. Trend lines are included for visualization purposes. (*c–h*) Species and age-related differences in the organization of the MR. Examples of flat-mounted lens epithelia from bovine (*c*), mouse (*d*), rat (*e*), rabbit (*f*), a 22-year-old human (*g*) and a 88-year-old human (*h*) stained with DAPI. In all cases, the MR of the lens epithelium is very apparent, but the human is the least well organized. Scale bars, 10 μm.

Firstly, the distance from the pole to the start of the peak in cell density was defined mathematically by drawing two lines in the plot of cell density versus radial distance (figure 2*a*); one was the best line fit in the central region of the CZ, where cell density was approximately invariant with position. This was a baseline. The other line was defined by the gradient through the data mid-way between the baseline level and the level at the peak in cell density at the lens periphery. The distance from the pole to the intercept of the lines defines the distance *r*_CZ_ as shown in figure 2*a*. This was straight-forward for bovine, human, rat and rabbit data. The human data lacked a well-defined peak in cell density and at this stage it is unclear whether this is a feature of human lenses *per se* or is due to the selected age range. The final and indeed highest valued data point was therefore taken to be the peak value. Defining the baseline for the mouse data presented some minor issues as discussed in the electronic supplementary material.

With these mathematical definitions, we could derive two dimensionless variables and therefore compare lenses of different sizes. The dimensionless variable *R* is the radial position *r* divided by *r*_CZ_; clearly *R* = 1 at *r*_CZ_. Secondly, *N* is the ratio of the cell density *n* at the point *r* to the average cell-density value in the central region of the CZ, *n*_0_. We also defined the maximum value of *R* at which cell density ceased to be approximately invariant with radial position as *R*_p_.

Data plotted in dimensionless form for bovine, young mouse (three months), rat, rabbit and human lenses are shown in figure 2*b*. The variation of dimensionless cell density *N* with dimensionless distance *R* for bovine, rat and rabbit gives rise to essentially one curve thus demonstrating species-independent behaviour for these lenses. The data for the mouse are similar, but with a slightly smaller peak in *N* at the periphery of the lens epithelium. This is attributed to an overestimate of the cell density in the region of the anterior pole for the reasons discussed (see the electronic supplementary material). The data for the adult (20–30 years old) human lens show a different trend with no obvious peak at the periphery, rather just a steady increase in cell density. This may be because the human lens data are for mature adults, whereas the animal lenses are from adolescents by comparison. Also the adult human data had *R*_p_ close to 0.5 while the values of *R*_p_ for all other species were very close to 0.8.

The absence of an apparent drop in cell density at the very periphery of the human lens epithelium is attributed to the short length of the MR compared with other species reported here (figure 2*c–h*). Comparing figure 2*g* (22-year-old human) with the other lenses (figure 2*c–f*) and also with figure 2*h* (88-year-old human), it appears that (i) the human lens peripheral region is the least well organized; (ii) the length of the MR for young adult human lens is the smallest despite its size, it being the second largest after the bovine lens; and (iii) there is a still further loss in the organization of the MR in the 88-year-old human lens.

### 3.6. Variation in epithelial cell density profile with age

**Figure 3.**
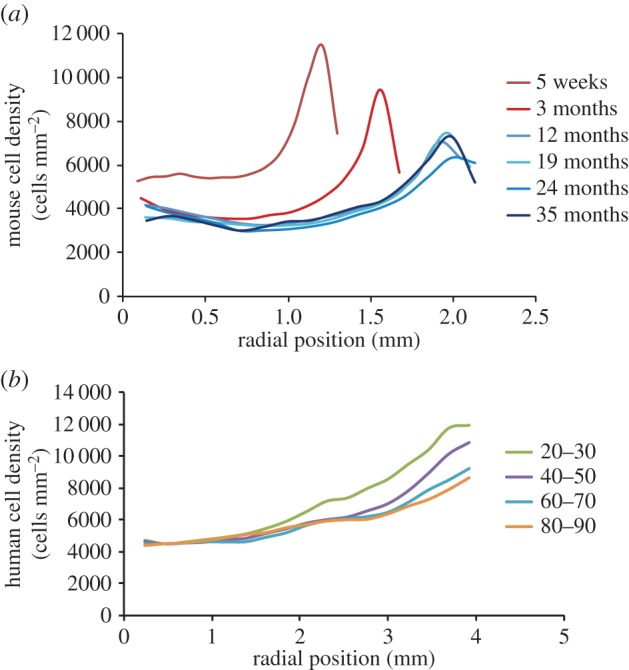
Variation of measured cell density with age in the lens epithelium of mammals. (*a*) Line plots for mouse lenses of different ages. (*b*) Line plots for human lenses of different ages.

To emphasize the evolution of cell density with age, the data for mouse and human lenses are shown as line plots (figure 3*a,b*). The measured cell densities were plotted against actual distance from the anterior pole in order to track the change in both lens size and epithelial cell density. Comparing the data for mouse lenses of different ages (figure 3*a*), there is an abrupt peak for young mice as found in other young mammals (figure 2*b*). This peak broadens with increasing mouse age (figure 3*a*). A distinct peak in cell density similar to that seen in the animal lens data is absent from our human data (figure 3*b*). In the four age groups of adult (more than 20 years) human lenses, the cell-density maxima in the periphery (1.38 mm in length) consistently declined with age (figure 3*b*). Significant differences between lens cell density profiles for the age groups 20–30 versus 40–50, 20–30 versus 60–70, 20–30 versus 80–90, and 40–50 versus 80–90 were observed (*p* < 0.05); the highest cell density of the oldest group had dropped by approximately 25% compared with the youngest group's. We note that the cell density maxima of the youngest human lenses were close to that of the bovine lenses (less than 30 months).

## 4. Model development and validation

### 4.1. Overview

Our OPT model was developed from first principles as set out in §4.2 and resulted in a ‘pull-through’ term being introduced to reflect the processes governing lens fibre cell formation from lens epithelial cells. The biological justification is presented later, but suffice to say that this term reflects the complex cell biological events accompanying the formation of lens fibre cells at the lens periphery, particularly in the MR and within the lens modiolus and fulcrum at the lens equator. We developed a model using a very simple measurement—cell density—and distinct morphological features, e.g. MR and the anterior pole in the CZ to define the physical boundaries of the epithelium. The governing equations for the cell-density profile were obtained by solving differential equations representing the cell population balance across the lens epithelium. One might term them as being conservation of species equations but due to the distinctive nature of the problem they were established from first principles. In common with Chung & Vafai [37], our conservation of species equation includes a production/proliferation term. We demonstrate that we can successfully model the cell density (which is an area density, i.e. cells mm^−2^) to give a distinct peak at the lens periphery in line with the experimental data for the species concerned. The ratio of two key parameters in our OPT model, one for the distal part of the CZ, the other for the peripheral region with the cell-density peak, which we assign as the GZ, correlated very well with the measured gradient of the lens morphogen FGF-2 as explained in §4.3.

**Figure 4.**
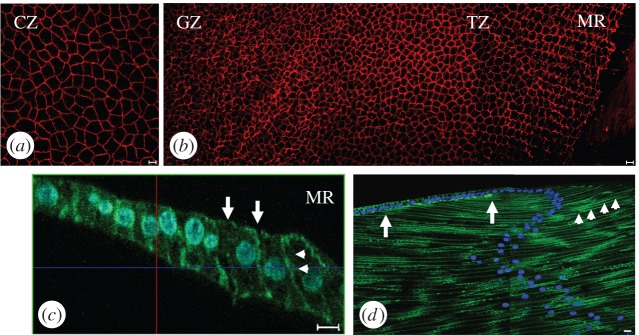
Cell shape and cell organizational changes across the bovine lens epithelium. (*a,b*) Cell profiles in CZ, GZ, TZ and MR of the flat-mounted bovine lens epithelium. Using the apical plasma membrane marker ZO-1 (red channel), the cells in the CZ (*a*) have the largest surface area (see electronic supplementary material, figure S1*a*). At the MR (*b*), the lens cells aligned into columns. Cells in the MRs have a hexagonal profile. Each column in the MR is offset by half a cell width to allow the interdigitation of neighbouring columns. (*c*) Actin staining in the TZ/MR is located mainly on the lateral (arrowheads) and apical (arrows) cell membrane in these elongated cells. Cell nuclei have been DAPI stained (blue channel) locate toward the apical ends of the lens cells. (*d*) N-cadherin (green channel) is concentrated along the lateral plasma membranes of lens fibre cells (arrows). It is also concentrated at the interface between the apical ends of epithelial cells in the TZ/MR and the most recently formed fibre cell (arrowheads). The concentration of N-cadherin between the two arrows at the interface between the apical ends of apposed epithelial and fibre cells identifies a region, which we interpret as the lens modiolus and fulcrum.

In §4.4, we considered how to model the decrease in the cell-density profile from the peak to the reduced density in the MR and the immediately adjacent TZ at the lens periphery. The reduction in cell density is *greater* than the minor change that would be predicted from a consideration of the increasing value of the circumference with radius. There is an *active* reduction in cell density and we present experimental data showing how cell–cell interactions are different within this region of the lens epithelium. For example, we highlight the formation of N-cadherin homotypic junctions between the apical ends of the epithelial cells and their apposed lens fibre cells (figure 4).

Finally in §4.5, the measured changes in cell density that accompany lens ageing in humans were predicted by the OPT model. For the mouse data, the model also reflected the trend with age. Overall validation was achieved with cell-density measurements in the ageing mouse (one month–2 years) and human (20–89 years) lens epithelia.

### 4.2. Ordered pull-through model for central and germinative zones

At steady state the change in cell density with time is negligible and we assume that the positional variation in cell density (*n*) is determined by the balance between net cell gain and the change caused by the demand to continuously replenish the epithelial cells lost from the epithelium to form new fibre cells.

The epithelium is neither a hemisphere nor a disc, but it covers a flattened oblate hemispheroid. We employed a two-dimensional cylindrical polar coordinate system with the origin at the pole of a flattened lens epithelium (schematic, figure 1*c*). As the data are derived from flat-mounted lenses, no transformation from three dimensions to two dimensions is required. Axial-symmetry can be reasonably assumed and the dependency upon angle is thus eliminated. The dependency of cell density upon position is then a function of the radial distance only as indicated in figure 1*c*.

Consider an annulus at a general radial distance *r* from the pole, of radial width d*r*. Within this elemental annulus the area is 2*πr*d*r*. The net gain of cells (number per unit time) in a given elemental area is assumed to be proportional to the local number of cells, which is the product of the cell density and area. By labelling the proportionality constants as *α* for the distal part of CZ and *β* for the GZ one obtains:

For distal part of CZ:4.1
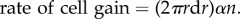


For GZ:4.2
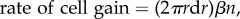
*α* and *β* are *per capita* cell gain rates, which are typically a number fraction (or number percentage) per day where *n* is the local cell density that varies with *r*.

The rate of cell gain within any annulus is balanced at steady state, by the difference in the migration of cells across the mathematical boundaries of the annulus at *r* and *r* + d*r*. As the lens continues to grow throughout life, cell migration is required. We assume that the movement across a boundary is given by a function dependent on the length of the boundary and the cell density at the boundary. The expression for the net passage is:4.3
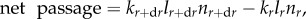
where *k*_*r*_ and *k*_*r*+d*r*_ represent the pull-through term at *r* and *r* + d*r*.

Now assuming that pull-through is spatially invariant for a given cell density:4.4
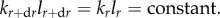


As4.5
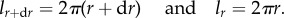


It follows from (4.5) and (4.6) that4.6
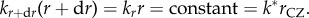
The characteristic radius *r*_CZ_ has already been defined in the §3.5. The term k^*^ is a spatially invariant pull-through parameter. This pull-through factor reflects the complex cell biological processes that orchestrate the ordered exit of the differentiating cells from the lens epithelium into the body of the lens.

Remembering that the constant in equation (4.6) is k*r_CZ_ and combining equations (4.1), (4.3) and (4.6), the differential balance for CZ becomes4.7
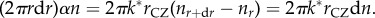
Equation (4.7) represents the balancing of the rate of net cell gain (number per unit time) with the difference between the number entering and the number leaving the annulus per unit time.

Hence4.8
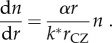
Now define *N* = *n*/*n*_0_, where *n*_0_ is the cell density in the polar region of CZ and *R* = *r*/*r*_CZ_, where *r*_CZ_ is the value of *r* at the boundary of CZ and GZ (as defined mathematically previously). Note, *n*_0_ is written later as *n*_CZ_ when datasets for particular age groups are considered, and *n*_0_ is used as the reference density for a mean value of various *n*_CZ_s when considering, for instance, adult mice of different ages. Integration of equation (4.8) gives4.9
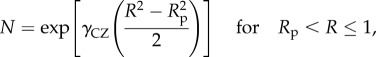
where4.10
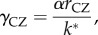
for *R* < *R*_p_, *N* = 1. The value of *N* at *R* = 1 is defined as *N*_CZ_. So for *R* > 1, and again integrating equation (4.8), one obtains for GZ4.11
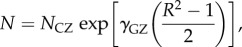
where4.12
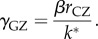
The terms *γ*_CZ_ and *γ*_GZ_ are dimensionless ratios representing the relative strength of the net cell gain to pull-through. The dimensions of *α* and *β* are per unit time; the dimensions of k^*^ are length per unit time. The terms *α* and *β* are related to, but not identical with, the proliferation or mitotic index (*I*), which is defined for a given area as the number of cells in the cell cycle divided by the total number of epithelial cells. Now as noted earlier *α* and *β* are *per capita* cell gain rates. If the cell cycle takes *T* hours to complete, and *I* (for GZ) is expressed as a percentage then the relationship to *β* (assuming minimal apoptosis) is4.13
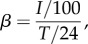
where the units of *β* are per day. For example, if *I* is observed to be 0.5% in GZ and *T* is 40 h (40 h selected arbitrarily), then *β* has a value of 0.003 *per capita* per day (i.e. three per 1000 cells per day).

### 4.3. Validation of ordered pull-through model with experimentally determined cell density as a function of distance from the central zone pole

**Figure 5.**
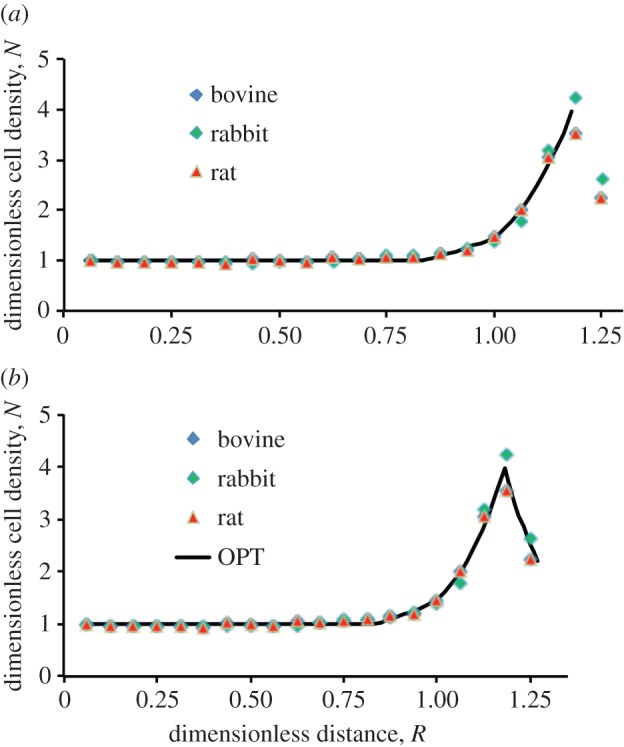
Comparison of the ordered pull-through model with the experimentally measured data. (*a*) Comparison of the model for lens epithelium (solid line) with the experimentally measured data (symbols). (*b*) Comparison of the ordered pull-through model for lens epithelium with experimental data obtained for flat-mounted bovine, rabbit and rat lenses.

The trend in lens epithelial cell density from the anterior pole to the MR was very similar for mouse, rat, rabbit and bovine lenses. The data were collected from relatively young animals. The cell density remained constant at the anterior pole, increased modestly in the distal CZ then quickly and dramatically peaked at a distance coincident with the GZ (figure 1*e*), declined to a terminal density at the MR (figure 2*a*) and including the TZ [9,11,38]. This trend (figure 5*a*) is common to several different mammalian lenses and is evidence that a single mathematical model applicable to all mammalian lens epithelia would be biologically relevant. For each mammal, best-fit parameters for the ratio *β*/*α* and *γ*_GZ_ were determined. It was found that the former ranged from 2.05 to 2.25 and the latter from 4.5 to 5.50. The fit in figure 5*a* was obtained with *β*/*α* of 2.16 and *γ*_GZ_ of 5.13. The fact that a single pair of values fits for differently sized lenses is important. The implication is that within the lens epithelium there is biological scaling to accommodate the different sized lens found in different animals. Interestingly, the ratio *β*/*α* correlates with the FGF-2 levels bound to the inner surface of the bovine lens capsule [34]. FGF-2 levels at the lens equator were double that found at the anterior pole, which accords with our computed *β*/*α* ratio of 2.16. FGF-2 regulates both cell proliferation and differentiation [39].

### 4.4. Ordered pull-through model for the transitional zone

When lens cells leave the epithelium, there are three important observations. Firstly, Ki-67 positive cells are concentrated in the GZ, rather than in either the TZ or MR of the peripheral region (figure 1*e,f*; [8,11,32,40]). Secondly, there is an ordering of the cells within, and between each of the individual MR as cells adopt a hexagonal profile. Their interdigitation requires offsetting neighbouring rows by a half-cell width (figure 4*b*). Thirdly, a modiolus and fulcrum is established via cell–cell interactions between the apical ends of epithelial cells immediately adjacent to the MR (figure 4*d*) and the newly formed fibre cells so as to maintain the geometrical order that accords with lens function [3]. Where there is a very clear boundary as in figure 2*c–f* for bovine, mouse, rat and rabbit lens, we suggest there is a switch from one set of conditions to another. Then mathematically it is assumed that at the distal, more equatorial margin of the GZ, there is a switch from one status to another, i.e. from one where cell proliferation and pull-through are determining *n* to one where the rate of decrease in *n* is balanced by cell pull-through into the lens mass. Therefore, for the cells between the GZ and MR, i.e. the TZ cell population [36], the differential balance is4.14
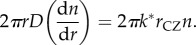
Hence4.15
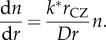
For *R* > *R*_peak_, one obtains4.16
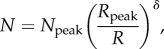
where4.17
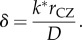
The term *R*_peak_ is the value of *R* at the peak of the profile and *N*_peak_ is the value of *N* at that point. The term 1/*δ* should be viewed as a dimensionless gradient coefficient. We emphasize that the underlying mechanism is not assumed to involve diffusion; equation (4.16) simply reflects the rate of decline in *n* within the TZ and MR with the value of *δ* indicating the strength of this change. Our hypothesis is that this decline in cell density is linked to the changes in the cell–cell interactions in the TZ and MR and their apposed lens fibre cells in the modiolus and fulcrum of the lens. The value of *δ* is proportional to the pull-through parameter k^*^ and depends upon the cell–cell interactions in this region. An alternative hypothesis is that the cells simply autonomously increase their volume and surface area, but unlike the pull-through hypothesis the mechanism for this is unclear.

When the cell-density measurements in the TZ and MR plateau as seen for the adult human lens (figure 2*f,g*), this changeover from one set of conditions to another (as assumed above) does not occur. Then instead of a decline in cell density, *n*, a plateau may be reached as seen for our human lens data.

We observed a concentration of N-cadherin at the apical ends of those cells immediately adjacent to the MR (figure 4*b–d*), where there is contact with the apical ends of lens fibre cells. The lens epithelial cells are much longer in this region (figure 4*c*; electronic supplementary material), coupled with the concentration of N-cadherin at the apical interface between epithelial and fibre cells, it is reasonable to expect increased cell–cell interactions that would contribute to increased cell adhesion. Indeed actin filaments are enriched at these points of cell–cell contact (figure 4*b*). The fact that one of the complications in cataract surgery is the removal of lens fibre cells from the lens equator provides circumstantial evidence of the increased strength of the cell–cell interactions at the equator as compared with other parts of the epithelium [41]. Quantitative electron microscopy of the epithelial–fibre cell interface confirmed the presence of many adherens junctions between the GZ/TZ epithelial cells and elongating fibre cells in contrast to central epithelial cells and their apposed fibre cells [23,42].

The OPT model is completed by incorporating equation (4.16). This then gives complete modelling of the lens epithelium from CZ through to the MR, for those cases where there is a distinct peak in cell density at the GZ. Figure 5*b* provides validation of the full OPT model. Given the limitation to resolve clearly the GZ, TZ and MR cell density for the human lens, the TZ and MR region has not been included in the model for aged human lenses, but an excellent match between the observed and predicted cell densities is still obtained (see §4.5).

### 4.5. Accounting for ageing effects on the cell-density profiles in the epithelia of mouse and human lenses

The model has dimensionless parameters *γ*_GZ_ and *γ*_CZ_ whose ratio is *β*/*α*. In the absence of evidence to the contrary, this ratio was assumed to be invariant with age and a simple equation was developed for the decline of *γ*_GZ_with time. The available data on the evolution of the profiles with age were for mice and adult humans. It was found that the *β*/*α* ratios fell either side of the single ratio established for adolescent mammals (bovine, rat and rabbit) being 3.0 for mice and *ca* 1.0 for human. The deviation from *ca* 2 might be due, at least in part, to age.

**Figure 6.**
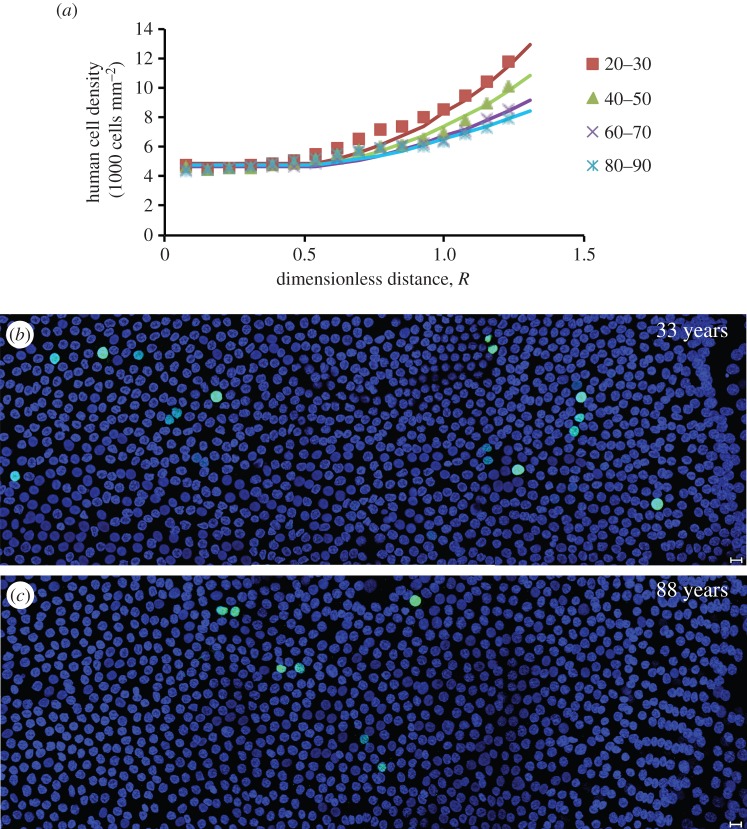
Modelling of the ageing of the human lens. (*a*) The model tracks the evolution of the human cell-density profile as measured in lenses of different ages. (*b*,*c*) The age-dependent decline in human lens epithelial cell proliferation. Representative images from flat-mounted human lens epithelia probed with the cell proliferation marker, Ki-67 (green channel) and DAPI (blue channel). Note that the 33-year-old lens (*b*) has more proliferating cells than the 88-year-old lens (*c*). In both cases, the GZ and MR are located on the right of the image. Scale bars, 10 μm.

Simple equations expressing the change in *βr*_CZ_/k* with age were obtained for mouse and human. From knowledge of both *βr*_CZ_/k* (i.e. *γ*_GZ_) as a function of time and *β*/*α* (taken to be constant), the profile within the mathematically defined CZ and GZ could then be predicted as shown in figure 6*a* for the adult human lens epithelium. The variation of the dimensionless parameter *γ*_GZ_ with age is similar for adult human and older mice; it declines steadily from 2.0 ± 0.1 to 1.1 ± 0.1. Our proliferation data (figure 6*b,c*) evidence that cell proliferation declined with age.

**Figure 7.**
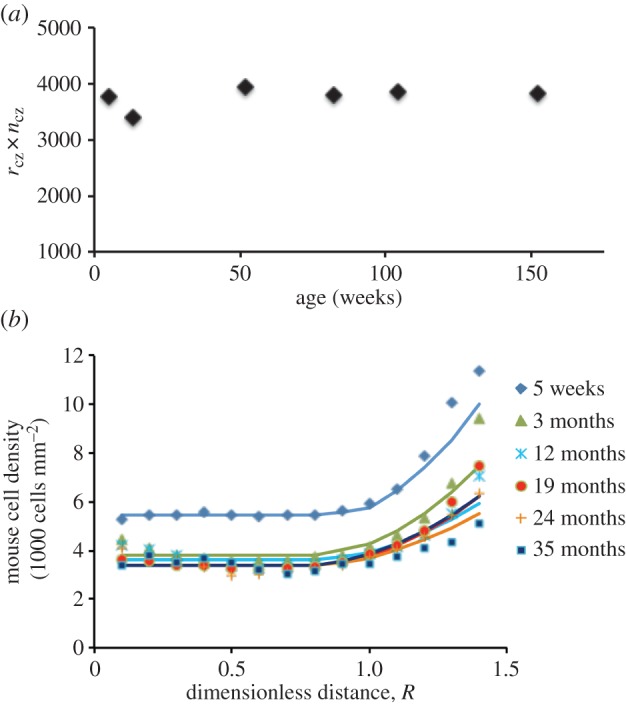
Modelling of the ageing of the mouse lens. (*a*) The product of *r*_cz_ and *n*_cz_ (i.e. *r*_cz_ × *n*_cz_) is a constant. (*b*) Validation of the OPT model using mouse data.

Our OPT model can also account for the overall trend in the age-related mouse data. For mice, figure 7*a* shows that the product of density, *n*_CZ_, and *r*_CZ_ is constant. This does not imply that the cell mass or the total number of cells in the lens epithelium is constant as area increases with 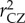. The observation that *n*_CZ_ × *r*_CZ_ is constant does, however, allow one to predict *n*_CZ_ at various ages given information on eye size as a function of age. This gives one a robust equation for the evolution of *n*_CZ_ with age; determining the evolution of cell density in CZ is the first part of our model to account for the effect of ageing. This observation that *n*_CZ_ × *r*_CZ_ is constant is consistent with the observation that the surface area of the epithelial cells in CZ increases with age [23]. The second part of the model is a relationship for the decline of *γ*_GZ_ with age. Together they can model the age-related changes in cell density (figure 7*b*).

## 5. Discussion

### 5.1. A single mathematical model to explain cell-density distributions for lens epithelia from different mammals

We propose the first mathematical model to account for the cell-density pattern of the mammalian lens epithelium. The model captures the lens' epithelial cell organization and is independent of the size differences between lenses of different mammals. From the variation of cell density with age it was shown for both mouse and human lenses that the ratio of average cell density of the lens periphery to that of the CZ decreases with increasing age.

As the age-related changes are relatively slow, quasi-homeostasis of the lens epithelium can be assumed. The OPT model balances cell pull-through due to fibre cell formation with net cell proliferation. The OPT model has three dimensionless parameters, *γ*_CZ_, *γ*_GZ_ and *δ*. The ratio *γ*_GZ_/*γ*_CZ_ is identical to *β*/*α* and produced a value equivalent to the measured FGF-2 gradient [34]. If the ratio of *β*/*α* is taken to reflect the ratio of FGF-2 then the OPT model can predict the morphogen gradient ratio between the central (CZ) and equatorial (GZ, TZ and MR) capsule. For bovine, the ratio of the *per capita* division rate for the CZ to that in the GZ was found to be equal to the experimentally measured bovine FGF-2 ratio [34]. Furthermore, the same *β*/*α* ratio was also found to hold (within 20%) in the modelling of the other species (e.g. rat, rabbit and mouse). For relatively young mammals, the OPT model is essentially species independent with common values of *R*_p_, *β*/*α* and *γ*_GZ_. The human data, albeit for adults, shows a different value of *R*_p_ and a lower peak in *N* compared with the young mammals.

### 5.2. Towards an understanding of age-dependent growth of the lens epithelium

In developing the full OPT model it was observed that the product of cell density, *n*_CZ_, and *r*_CZ_ is constant. This quantifies the evolution of cell density in CZ and explains why the surface area of CZ cells increases with age (e.g. [23], because cell density in the CZ is a function of *r*_CZ_).

From the definition of *γ*_GZ_ and equation (4.13), it is readily shown that5.1
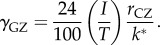
From electronic supplementary material table S1, one can note that the proliferation index (*I*) halves approximately in human during advancing adulthood. The ability of lens epithelial cells to respond to morphogens such as FGF-2 declines with age [21]. Now as noted above *γ*_GZ_ halves from around 2.1 to 1.1 and this is consistent with an approximately constant k^*^, an approximately constant value of time for mitosis *T* and a halving of the proliferation index. If *T* were to increase slightly with age (i.e. mitosis rate were slower) this might well be balanced by k^*^ decreasing slightly with age such that these two effects cancel out. The biological consequence of this decrease is the changes in the TZ and MR of older human lenses (figure 2).

From equation (5.1), it is clear that if values for *γ*_GZ_ and information on *I*, *T* and eye size (hence *r*_CZ_) are known, then one can estimate k^*^. The fact that in electronic supplementary material, table S1, the proliferation index for rabbit is different from bovine (of similar age) does not contradict the observation above that there is a species-independent value of *γ*_GZ_. This dimensionless parameter is dependent upon the proliferation index, the values of *T* and k^*^. Knowledge of the relevant values of the mitotic time, *T*, would enable *β* to be calculated. Then species-specific values of k^*^, the spatially invariant pull-through parameter, could be calculated and its value would provide a measure of the observed differences in the cell organization in the MR and adjacent TZ of different species (figure 2*c–g*). Variation in k^*^ as a function of age with one value for early life and another for adulthood could explain the observation that in some mammals growth has a biphasic mode as in the case of the human lens [2].

One should note that three dimensionless parameters suffice to define the OPT model: *γ*_CZ_ for the outer part of CZ, *γ*_GZ_ for cell-density peak, i.e. GZ and *δ* for the TZ and MR; the respective equations are (4.10), (4.12) and (4.17). All three dimensionless parameters include k^*^ which has been labelled as the pull-through parameter, because it was seen to control the fraction of cells passing through the TZ and MR.

### 5.3. Cell–cell interactions in the lens epithelium that contribute to the ordered pull-through model

The dimensionless parameter *δ* in the OPT model is dependent upon k^*^, the pull-through parameter. This parameter reflects the cell–cell attraction component in the peripheral region of the lens at its equator. Here we observed the concentration of N-cadherin at the epithelial–fibre cell interface. Attractive forces between neighbouring cells in the plane of the epithelium will also change in this region because of the significant increase in the aspect ratio of the epithelial cells in the lens periphery. Lens-specific deletion of N-cadherin in mice alters actin distribution and induces fibre cell elongation defects [43]. The disruption of apicalapical cell interactions between epithelial cells and their apposed fibre cells also stimulates epithelial cell proliferation [5], in response to the relaxation of the spatial constraints that regulate cell proliferation [44]. Cell proliferation in the lens epithelium is also vimentin dependent [45]. Vimentin is associated exclusively with N-cadherin/γ-catenin junctions in differentiated fibre cells and may contribute tensile strength to maintain the hexagonal profile of MR and fibre cells [46] as the differentiating epithelial cells traverse the modiolus. Taken together with the observations in §4.4, it is clear that there is a considerable body of evidence to support an equation reflecting the interactions of, and ordering of, epithelial cells in the MR, the adjacent TZ, the lens modiolus and fulcrum and the apposed lens fibre cells.

Equation (4.16) is fully justified where the boundary between GZ and TZ/MR is distinct and associated with a changeover from one set of conditions to another. Under these conditions, the rate of decrease in *n* is simply balanced by cell pull-through into the lens body via the MR. However, as shown in figure 2*g,h*, a clear boundary as defined by the cell-density measurements in the GZ and TZ/MR appears not to exist in adult human lenses. The boundary is weak in the human lens even at age 22 years (and absent at 88 years old). While boundaries are a mathematical requirement generic to the model's present construction if subsequent molecular and cell biological markers were to provide more precise spatial definitions of the GZ, TZ and MR they could easily be incorporated into the OPT model.

## 6. Concluding remarks

In this work, we developed the first mathematical model of cell-density distribution in the lens epithelium of animal lenses. From our experimental data, we showed that there are species-independent profiles for adolescent mammals, if the data are normalized based upon the size of CZ. The increasing cell density was observed to start at *R* = 0.8 (where *R* is a normalized radial distance to account for the difference in lens size between species) and to be rapid above *R* = 1.0 reaching a peak at *R* = 1.2. This was successfully represented by the OPT model that balances the pull-through due to fibre cell formation with net epithelial cell gain. The full form of the OPT model has three dimensionless parameters, *γ*_CZ_, *γ*_GZ_ and *δ*. The ratio *γ*_GZ_/*γ*_CZ_ is identical to *β*/*α* and this coincided with the measured FGF-2 gradient [34]. It is noted that the dimensionless parameter *δ* is required only for the lens region between the peak in cell density and MR and is only applicable if there is a clear boundary between the peak density and the density at the MR, i.e. the most peripheral lens region. This term was inapplicable in our analysis of ageing human and mouse lenses and so these validated models needed just two parameters.

Regarding the evolution of cell density of the epithelium with age, a key observation was that the product of cell density in the CZ zone and the radius of the lens is approximately constant for mouse. Cell proliferation in the CZ is repressed by contact with the underlying fibre cells [5], but FGF-2 will drive proliferation in the GZ via MAPK1 [47], although proliferation declines with age in the human [21]. With an expression for the reduction of *γ*_GZ_ with age, we then used the OPT model to successfully predict ageing of lens epithelia in both mouse and human, the latter being confined to adulthood. The dimensionless *γ*_GZ_ parameter was found to halve during adulthood and reflects the decrease in the net gain parameter *β* with age.

The scaling of tissues in differently sized organisms is an important biological question [48]. Scaling requires modulators/morphogens to be recognized by all cells and that at least one of these that determines tissue size is fixed so that the distribution of other modulators can then be scale invariant [48]. In the lens epithelium, evidence of this key reference point is the cell-density peak and the concentration of a key morphogen, such as FGF-2, at the lens equator [34]. Such morphogens are likely candidates to determine scaling lens size in the different mammals. All cells in the lens epithelium respond to this growth factor [39] and it is bound to the surface of the inner lens capsule in a concentration-dependent fashion with respect to radial distance [34]. The influence of the capsule structure, diffusion and consumption rates of such morphogens will be important and here LDL transport across the arterial wall could be a useful model paradigm [37]. The OPT model, we have developed is independent of lens size for the mammals we analysed and evidences its scaling property.

## Supplementary Material

Supplementary Material

## Supplementary Material

Supplementary Material Figures

## Supplementary Material

Supplementary Table
