# Supplementary material for: A dimensionless ordered pull-through model of the mammalian lens epithelium evidences scaling across species and explains the age-dependent changes in cell density in the human lens
Source: J R Soc Interface. 2015 Jul 6;12(108):20150391. doi: 10.1098/rsif.2015.0391 (PMC4528606; doi:10.1098/rsif.2015.0391)
Supplement: Supplementary Material [file rsif20150391supp1.pdf]

Supplementary Material

There are four parts:
1. Comparison of flat-mounted 2D and 3D density data
2. Pipeline of Image Analysis
3. Bovine cell density and cross-sectional data
4. Comments on mouse data
5. Proliferation index in lens epithelium for different species

1. Comparison of flat-mounted 2D and 3D density data

We used a flat-mounting technique. This avoids the potential to distort the lens which may occur when whole intact lenses are held in a wedged holder prior to imaging and the associated risk of distorting images at points of high curvature eg the mouse lens. Epithelial cell density measurements may also be distorted during the transformation of a three dimensional structure into a two dimensional flat mount, a problem which becomes more of an issue the larger the curvature of the anterior lens surface. Whether or not projection is an issue for rat lenses was assessed by making measurements on an intact rat lens before and after dissection. This confirmed that the measured cell density was similar after dissection and after flat-mounting; our flat-mount measurements for the peak of GZ and for TZ cross-checked with those for the intact rat lens. See Supplementary Figure 1. It was concluded that for eye lenses equivalent in size to the rat or larger could be effectively analysed after flat-mounting. For the mouse lens, there are additional considerations; see section 4 below.

2. Pipeline of Image Analysis

DAPI labelled nuclei were counted using a Python-based package "Delineator" because commercial and free (e.g. ImageJ) packages were unable to reliably count cell nuclei and manual counting was inefficient for our sample sizes. The steps of image analysis were:

1) Initially a simple test was run to make an approximation on the average nucleus size and number of nuclei to guide the fitting parameters. The user drew a circle around a "typical" nuclei to provide an estimate of the average nuclei dimension (giving a radius *a)* and the image was then subject to a binary threshold to determine the approximate number of pixels involved in the nuclei in that image. Using the two figures the approximate number of nuclei (N) was thus determined.
2) The original image was convolved with a matched filter to highlight the centre of each nucleus. The filter kernel was square with sides of 2*a* and with a value of -1 except in a centered circle of radius *a*, where it was +1. This gives an intensity peak at the centre and helped to smooth high spatial frequency noise with each nucleus

3) Using the convolved image we then found the brightest group of four pixels, which was taken as the centre of the first cell nucleus. From this point we extended 12 equally spaces radial lines to a distance of 1.5 times *a* (the typical radius) in the convolved image. These lines will span from the centre of the nucleus to either another nucleus or empty space. We defined the edge of the nuclei as being the point on each radial line where the intensity fell to a value of 20% of the maximum to minimum intensity (again using the average of the four pixels at the centre).
4) A polygon was fitted to the 12 points.
5) The polygon was converted to a binary mask. This mask was then used to zero pixels with the polygon in the convolved image.
6) The process was then repeated from step 2 with the new image (with the first nucleus now removed). This loop was then repeated 1.2 times N (the initial estimate of the number of nuclei).
7) In order to remove small noise artefacts that might now have been counted the nuclei found were now examined and any found to be less than 75% of the size of the initial "average" nuclei were rejected. The remaining nuclei were then highlighted using a circle around each and the user could examine the resulting image to provide a visual inspection of the result.

Once the user had made the initial selection the process was rapid and thus it was easy to manually adjust the parameters to ensure all nuclei were counted. An average image contained around 250-400 nuclei.

3. Bovine cell density and cross-sectional data

Supplementary Figure 2 shows that average cell area measurements (in μm^2) across the bovine epithelium. These apparently decline gently within CZ from the anterior pole to the periphery. The density measurements (number per mm^2) however show a central plateau in the CZ region (Fig. 2A and supplementary Fig. 2B). The product of these two numbers is expected to be a constant. However there is no statistically significant incompatibility; note the large error bars for cell area measurement.

The cell density measurements indicate 3500 cells per mm^2 and appear incompatible with the area measurements, but again an area compatible with 3500 cells per mm^2 is within the standard deviation for the cell area measurements. We are confident that our measurement of cell density is reliable as well as automated. A similar package may be developed for cell area in the future.

4. Comments on mouse data

Internally warping (because the capsule is less elastic in some regions than others) was a potential issue for some of the data that were collected for mouse lenses. Some distortion of a lens epithelium during mounting may occur with more spherical lenses such as the mouse. Except for the five week-old mouse data, the other mouse data apparently showed density to be somewhat higher near the pole and then fall to a minimum before increasing again to a peak. For the five week-old mice, the eight data points from the pole had given an essentially constant value and so the equivalent eight points were used to define n_0, the average cell density in the central region of the CZ for the other mouse lens data.

The data in Figure 2B are similar to those obtained for bovine, rat and rabbit, but with a slightly smaller peak in N at the periphery of the lens epithelium. If, as we believe, the cell density in the region of the anterior pole of the mouse lens is overestimated, then n_0 is too high and N is as a consequence is underestimated. Whilst absolute values of cell density could be affected by lens geometry, the trend in the data collected for lenses from different aged mice is unambiguous.

5. Proliferation index in lens epithelium for different species

A comparison of the proliferation index in different species is given in Supplementary Table 1.
