# Supplementary material for: A dimensionless ordered pull-through model of the mammalian lens epithelium evidences scaling across species and explains the age-dependent changes in cell density in the human lens
Source: J R Soc Interface. 2015 Jul 6;12(108):20150391. doi: 10.1098/rsif.2015.0391 (PMC4528606; doi:10.1098/rsif.2015.0391)
Supplement: Supplementary Material Figures [file rsif20150391supp2.pdf]

The figures below relate to sections 1 and 3 of the Supplementary Material.

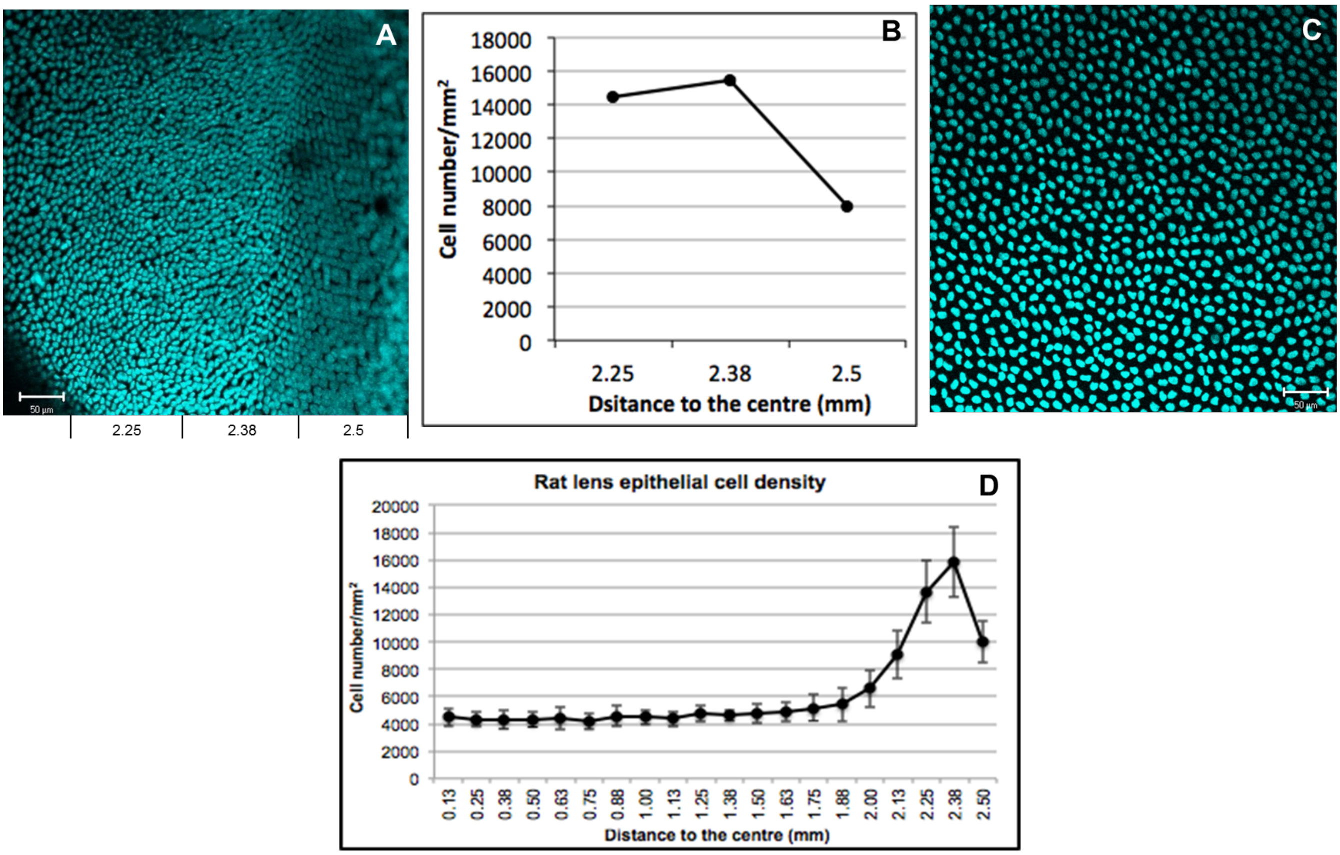

Supplementary Material Figure 1

Comparison of whole lens and flat mount technique: measurement of cell density in a whole rat lens. A 6 week-old rat was sacrificed and the whole eye removed. The lens was dissected from the eye and then incubated in medium 199 in the presence of the vital dye Hoechst 33342 for 30 minutes at 37^0C in an incubator prior to imaging. Hoechst 33342 was dissolved in DMSO and added to medium 199 to a final concentration of 1µg/ml. Lenses were imaged using a Zeiss LSM 510 microscope).

A. Representative image captured of Hoechst 33342 labelled nuclei at the lens equator of a freshly dissected rat lens.
B. Nuclear (cell) density measurement made from this image
C. Rat lens anterior pole stained with Hoechst 33342. The nuclear density in this region in the rat lens was measured as 5320 cells/mm^2.
D. Cell density profile for 6-8 week rat lenses measured using the flat mount technique. These data represent a total of X lenses, plotting the mean ± SEM

The measured cell densities in the intact lens agreed very well with the mean cell densities in the different regions of the lens. Scale bars in A and C = 50 µm.

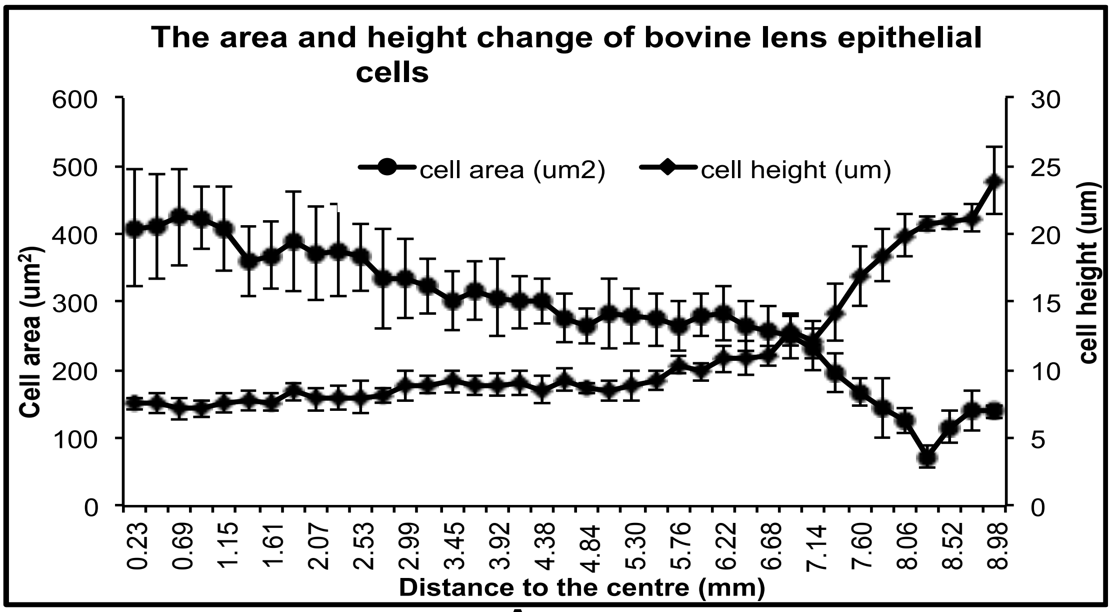

A

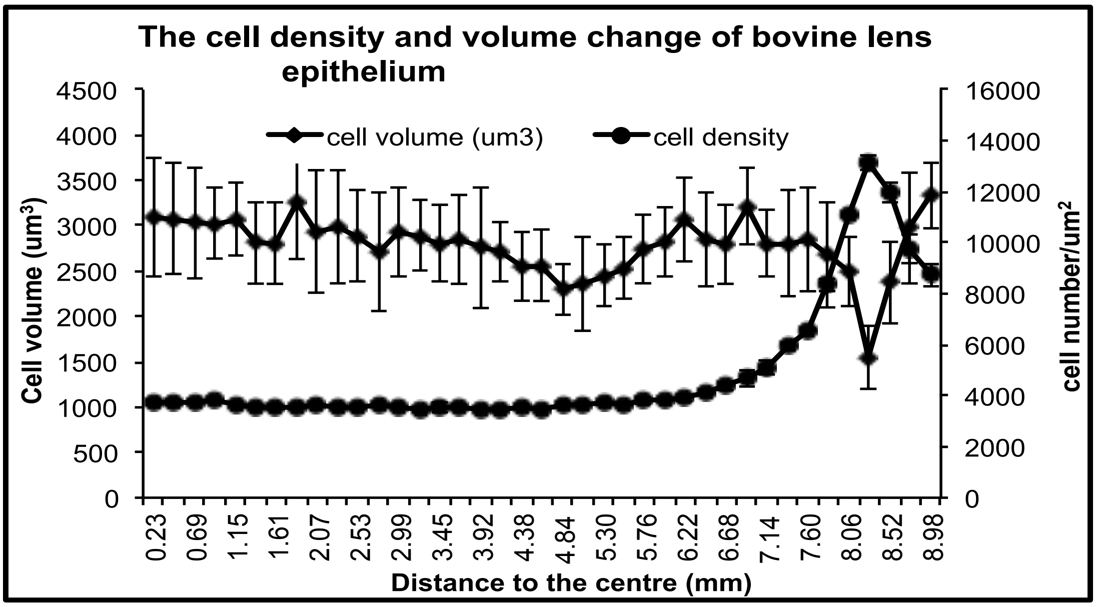

B

Supplementary Material Figure 2 Area and height (A), and cell density and volume changes (B) of bovine lens epithelium across flat-mounted bovine lens epithelia

The bovine epithelium flat-mounts were fixed in 4% (w/v) PFA for 20 minutes. After three washes in PBS, they were stained with phalloidin and DAPI (1 µg/ml) for 1 hour at room temperature. The whole-mounts were then rinsed three times in PBS and transferred to the microscope slides to make the slides. Images were taken with the Zeiss LSM 510 Meta confocal microscope. A series of images in a Z-stack were taken from the anterior pole to the MR. The cross section and longitudinal section of the cells in each image were saved for subsequent analysis. The cell height and cross-sectional area in each image were measured using ImageJ. The cell volume was calculated by multiplying the cell height by the cell cross-sectional area. At least 30 cells per image were randomly chosen for counting. Each data point is the mean ± SD.
