# Supplementary material for: A dimensionless ordered pull-through model of the mammalian lens epithelium evidences scaling across species and explains the age-dependent changes in cell density in the human lens
Source: J R Soc Interface. 2015 Jul 6;12(108):20150391. doi: 10.1098/rsif.2015.0391 (PMC4528606; doi:10.1098/rsif.2015.0391)
Supplement: Supplementary Table [file rsif20150391supp3.doc]

**Supplementary Table**

**Table 1** Proliferation index in lens epithelium for different species

| **Species** | **Bovine** | **Rabbit** | **Human (30-50yrs)** | **Human (80-90 yrs)** |
| --- | --- | --- | --- | --- |
| Sample number | 5 | 3 | 13 | 12 |
| Proliferation index % | 0.39±0.08 | 1.0±0.18 | 0.42±0.12 | 0.22±0.1 |
